# On the spectral changes of magna‐field photon beams used in total body irradiation and impact on dosimetry

**DOI:** 10.1002/acm2.70377

**Published:** 2025-11-18

**Authors:** Ahtesham Ullah Khan

**Affiliations:** ^1^ Department of Medical Physics, School of Medicine and Public Health University of Wisconsin‐Madison Madison Wisconsin USA

**Keywords:** in vivo dosimetry, Monte Carlo, reference dosimetry, total body irradiation

## Abstract

**Purpose:**

Accurate dosimetry for magna fields can be challenging when dosimeters exhibit energy dependence. The aim of this work was to use Monte Carlo (MC) simulations to calculate energy spectra of magna photon fields and use the spectral information to derive energy dependence correction factors for commonly used dosimeters.

**Materials and methods:**

Photon beams of 6 and 10 MV energies were simulated using the TOol for PArticle Simulation (TOPAS) MC code. Reference and magna field conditions were simulated with differing field sizes and source‐to‐surface distances (SSDs). Average photon energy and tissue phantom ratios (TPRs) were calculated as a function of depth in addition to photon energy spectra at various depths. Previously reported known energy responses of a Farmer ion chamber, LiF‐thermoluminescent dosimeters (TLDs), and alumina‐optically stimulated luminescent dosimeters (OSLDs) were extracted to calculate the energy dependence correction factor k_NR_ in magna fields.

**Results:**

The average photon energy decreased with depth, followed by an increase in the deepest regions due to the build‐up of scattered photons. At the phantom midplane, the average photon energy changed from 1.3 to 0.6 MeV for the 6 MV beam and from 2.3 to 1.1 MeV for the 10 MV beam with the transition from reference to magna field conditions. The energy dependence was found to be negligible for the ion chamber, up to 2.0% for the TLDs, and up to 4.6% for the OSLDs.

**Conclusions:**

Large spectral changes were reported between the reference conditions and the magna field conditions. The influence of beam softening in magna field was determined to be considerable for dosimetry using TLDs and OSLDs.

## 1 INTRODUCTION

Total body irradiation (TBI) aims to irradiate the entire patient in order to kill malignant cells in the bone marrow and suppress the immune system in preparation for a successful bone marrow transplant.^1^ Conventional TBI techniques rely on magna photon beams that encompass the entire patient placed at an extended source‐to‐surface distance (SSD) of over 400 cm. Modern TBI techniques utilize volumetric arc therapy (VMAT) at standard SSDs.^2^ Although VMAT‐TBI is expected to become more prevalent due to the dosimetric advantages it offers with more robust patient setup, most clinics still employ magna fields for TBI due to their simplicity. The scope of this work is limited to magna fields, that is, large photon fields at extended SSDs. Unlike standard SSDs, dosimetry in magna fields has much larger uncertainties and requires specialized equipment that is unavailable at most clinics.

The biggest challenge in performing accurate dosimetry in magna fields is the large field size of the radiation beam, requiring sufficient phantom size and radiation detectors with minimal stem/cable effects and energy dependence.^3^ Reference dosimetry in magna fields is typically performed using ionization chambers embedded inside a plastic or a solid water phantom. Therefore, the measured dose should be corrected using the ratio of mass‐energy absorption coefficients of water to phantom medium ${\left(\frac{\mu_{en}}{\rho }\right)}_{\mathrm{med}}^{\mathrm{water}}$. The calculation of such a ratio requires spectral information of the magna field, which is currently unavailable for high‐energy photon beams. Broad photon fields have increased phantom scatter, leading to softening of the energy spectra, which varies with depth.^4^ A study by Garcia et al. demonstrated differences in the measured tissue phantom ratio (TPR) at extended SSDs and inverse‐square‐corrected percent depth dose (PDD) curves measured at conventional SSDs.^5^ These differences are likely attributed to the beam softening effect expected at large field sizes. Energy dependence of the ionization chamber must be corrected using a beam quality correction factor due to the differences in the beam energy between the calibration and measurement beams. Besides reference dosimetry, in vivo dosimetry is performed using solid‐state detectors or metal‐oxide‐semiconductor field‐effect transistors (MOSFETs), which manifest significant energy dependence.^6^, ^7^ The availability of magna field energy spectra will allow the calculation of correction factors for these detectors. However, energy spectra of high‐energy photon beams are challenging to measure.^8^ Monte Carlo (MC) simulations provide an alternative method to calculate the spectral information of magna fields. However, such simulations require immense computational resources due to the large number of photons involved in the simulations. Modern computing resources have allowed such simulations, which can be used to derive correction factors for TBI dosimetry.

The work of Chofor et al. provided a method to calculate correction factors in various non‐reference conditions based on the energy spectra, mass energy absorption coefficients, and the relative response of the detector.^9^ The correction factor for non‐reference conditions, $k_{\mathrm{NR}}$, can be calculated by:

$$k_{\mathrm{NR}}=\frac{Y_{\mathrm{ref}}}{Y_{\mathrm{clin}}}$$

with

$$Y=\frac{\sum_{i=1}^{n}r\left(E_{i}\right){\left(\frac{\mu_{en}\left(E_{i}\right)}{\rho }\right)}_{\mathrm{water}}\varphi \left(E_{i}\right)E_{i}\Delta E_{i}}{\sum_{i=1}^{n}{\left(\frac{\mu_{en}\left(E_{i}\right)}{\rho }\right)}_{\mathrm{water}}\varphi \left(E_{i}\right)E_{i}\Delta E_{i}}$$

where 1≤i≤n are the bins corresponding to energy $E_{i}$, $r(E_{i})$ is the relative energy response of a given detector, $\Delta E_{i}$ is the bin size, ${\left(\frac{\mu_{en}\left(E_{i}\right)}{\rho }\right)}_{\mathrm{water}}$ is the mass energy absorption coefficient of water, and $\varphi (E_{i})$ is the fluence for a given energy bin. It is of note that the equation above assumes secondary electronic equilibrium in water. Given the spectral information for the reference and magna field conditions, the energy dependence correction factor can be derived for any detector with a known energy response. Such an initiative will improve the dosimetric accuracy of TBI treatments. Thus, the aim of this work was to employ MC simulations to calculate the energy spectra of magna photon fields and use the spectral information to derive energy dependence correction factors for commonly used dosimeters.

## 2 METHODS

All MC simulations in this work were run in sequential mode using the Open Science Grid (OSG) computational cluster.^10^, ^11^ Each run consisted of 3000 concurrent independent simulation jobs, which were combined at completion.

The TOol for PArticle Simulation (TOPAS) MC code was used in this work, which has been previously well‐validated for photon/electron transport physics.^12^, ^13^ Due to its high accuracy for multiple Coulomb scattering physics models, the *G4EMStandardOpt4* electromagnetic physics list was employed.^14^ Atomic de‐excitation physical processes were turned on for all simulations. The production thresholds, which determine the residual range under which the remaining energy is deposited locally, were set to 1 mm. The vendor‐provided IAEA phase space files were used to simulate 6 and 10 MV photon beams from a Varian TrueBeam commercial linac (Varian Medical Systems, Palo Alto, CA, USA).^15^ These energies are commonly used for magna photon fields for TBI treatments. Collimation jaws were explicitly modeled that were divergent with the field size. Simulations were performed under both reference conditions and magna field conditions. The reference conditions consisted of a virtual water phantom of 40 × 40 × 40 cm^3^ volume with an SSD of 100 cm and a field size of 10 × 10 cm^2^ projected at 100 cm distance from the source. Under 100 cm SSD, simulations were also performed for the 40 × 40 cm^2^ field size to compare the depth dose under magna field conditions with the inverse‐square corrected depth dose calculated under reference conditions. Magna field conditions were simulated with multiple water phantom sizes, including (i) 30 × 30 × 30 cm^3^ representing the size of standard solid phantoms commonly used in the clinic; (ii) 165 × 40 × 40 cm^3^ representing the size of an average patient; and (iii) 400 × 400 × 40 cm^3^ representing a near‐infinite phantom with full scatter condition. For each simulation, the phantom was placed at an SSD of 450 cm with the projected field size of 180 × 180 cm^2^ at the surface of the phantom. A Lucite spoiler of 1.5 cm thickness was placed 25 cm proximal to the phantom's surface. These conditions closely resemble the clinical conditions for TBI treatments with magna photon fields. PDD and average photon energy were scored along the central axis in voxel bins of 3 × 3 × 2 mm^3^ volume with 2 mm in the depth direction. To remove the impact of differing SSDs between TBI and reference conditions from the depth dose data, Maynard's F factor was applied to PDDs acquired with 100 cm SSD to compare with the TBI conditions. Additionally, PDDs were converted to TPR, with *d*_max_ as the reference depth, to further isolate the effect of attenuation from inverse square fall‐off. Photon energy spectra were scored at 0, 5, 10, 20, and 35 cm depths in voxel bins of 5 × 5 × 5 mm^3^ volume. These voxel sizes were chosen to maximize the computational efficiency of the simulations with minimal statistical noise. For each simulation, 200 billion histories were run to achieve a statistical uncertainty of <0.5%.

The energy dependence correction factor, $k_{\mathrm{NR}}$, was calculated for a Farmer‐type ionization chamber (NE 2571), LiF thermoluminescent dosimeters (TLDs), and alumina‐based optically stimulated luminescent dosimeters (OSLDs). These dosimeters were chosen due to their wide use in magna field dosimetry. The relative energy response, $r(E_{i})$, was extracted from previously published data.^9^, ^16^, ^17^ The reference (or calibration) response, $Y_{\mathrm{ref}}$, was derived using the energy spectrum calculated at 10 cm depth in reference conditions of 100 cm SSD and 10 × 10 cm^2^ field size. Using these data, energy dependence correction factors were calculated at 0, 5, 10, 20, and 35 cm depths in magna fields for both 6 and 10 MV photon beams.

## 3 RESULTS

**FIGURE 1:**
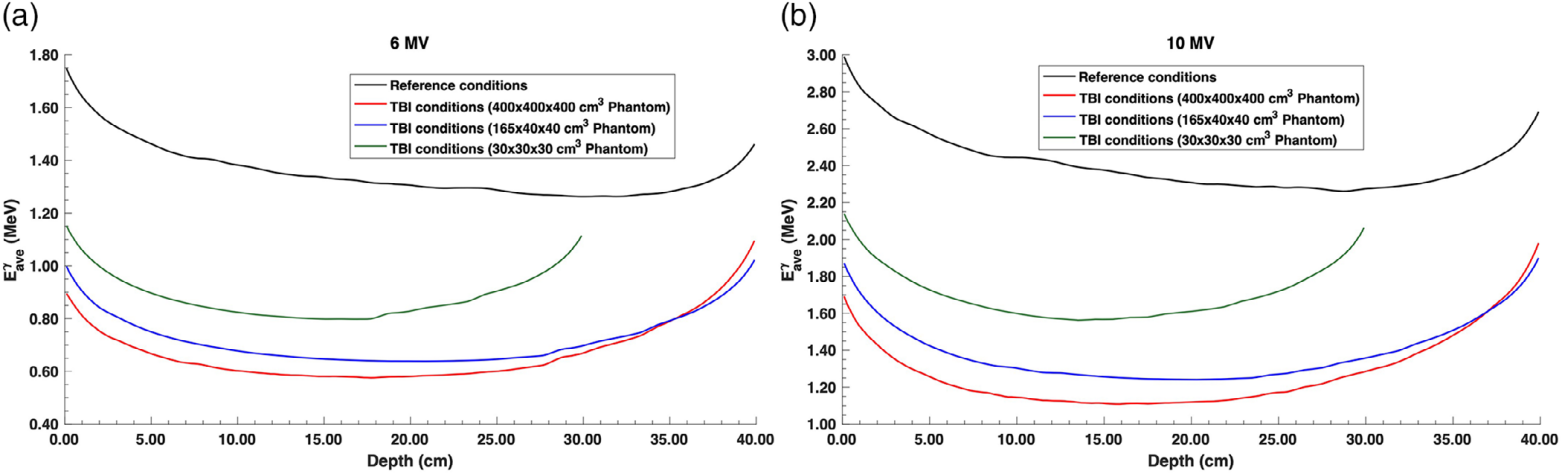
Average photon energy as a function of depth for both (a) 6 MV and (b) 10 MV photon beams under reference field conditions and magna field (TBI) conditions with various phantom sizes. TBI, total body irradiation.

The change in average photon energy as a function of depth is displayed in Figure 1. As expected, the average energy of the magna fields behaves as a typical high‐energy broad photon beam, where the mean photon energy decreases as a function of depth due to the buildup of low‐energy Compton‐scattered photons and increases again at deeper depths due to the beam hardening effect. The 6 MV beam was noted to be more impacted by the scattered photons compared to the 10 MV beam. With the transition from reference conditions to magna field conditions with near‐infinite phantom size, the average photon energy decreased from 1.35 to 0.67 MeV for the 6 MV beam and from 2.41 to 1.28 MeV for the 10 MV beam. Therefore, the 10 MV average energy was found to experience a larger spectral shift. The 10 MV magna field with near‐infinite phantom size was observed to have similar energy as the 6 MV reference field, with a difference of 0.24 MeV at 10 cm depth. With the decrease in phantom size, the average energy was found to increase due to the reduction of low‐energy scattered photons present in the phantom. The mean energy differences between the near‐infinite (400 × 400 × 40 cm^3^) and adult‐sized (165 × 40 × 40 cm^3^) phantoms were 0.047 and 0.108 MeV for the 6 and 10 MV beams, respectively. However, a large shift in average photon energy was noticed for the smaller 30 × 30 × 30 cm^3^ phantom, indicating the inability to achieve full scatter conditions.

**FIGURE 2:**
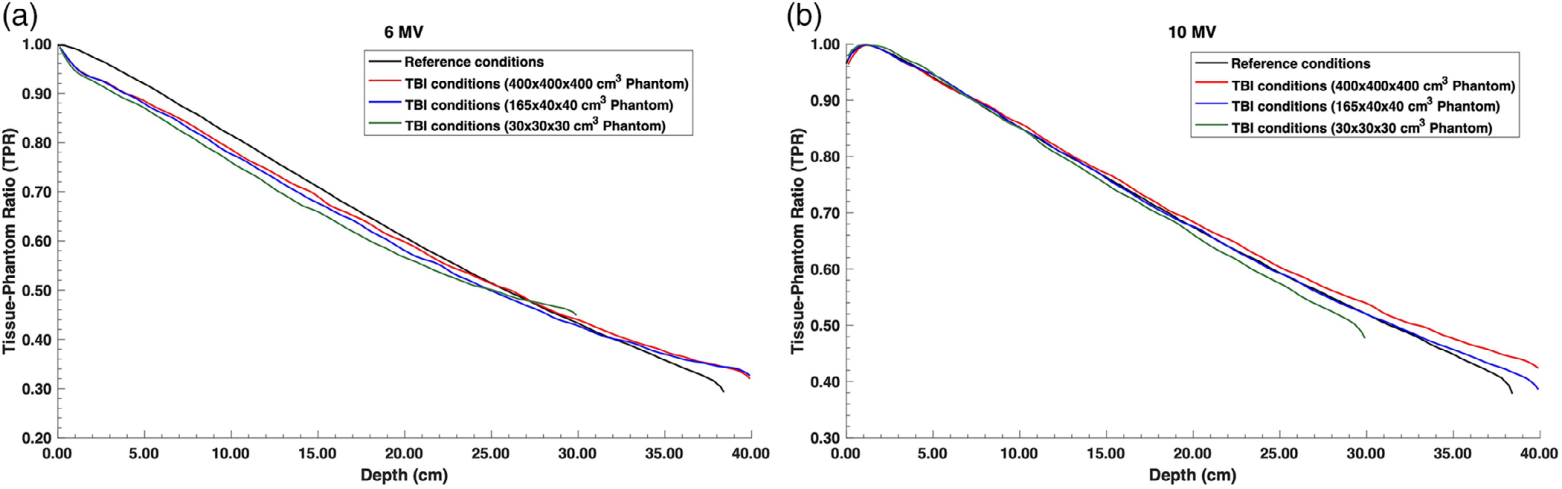
TPRs for both (a) 6 MV and (b) 10 MV photon beams under reference SSD conditions with a 40 × 40 cm^2^ field size and TBI conditions with various phantom sizes. The reference condition TPRs were shifted by 1.5 cm to account for the presence of the spoiler in the TBI conditions. SSD, source‐to‐surface distance; TBI, total body irradiation; TPR, tissue phantom ratio.

Figure 2 shows the TPR for both magna field and reference field conditions corrected for the differing SSDs. For the 6 MV beam, large differences were noted between the corrected reference TPR and near‐infinite phantom TBI TPR, especially in the shallow region, with a mean difference of 4% across all depths. These differences are likely due to the presence of a spoiler for the TPR condition while using a correction for the spoiler thickness for the reference TPR. However, better agreement was noted for the 10 MV beam between the reference TPR and TBI TPR with a mean difference of 2% across all depths. The mean difference in the TPRs between the near‐infinite phantom and adult‐sized phantom was calculated to be 1.5% for the 6 MV beam and 2% for the 10 MV beam, while these differences increase to 2.7% for both energies when TPR is compared between the near‐infinite phantom and the smaller 30 × 30 × 30 cm^3^ phantom. Interestingly, the TPR differences between the phantoms are more pronounced in the 5–20 cm depth range for the 6 MV beam and >20 cm depth for the 10 MV beam, likely attributed to the lack of backscatter.

**FIGURE 3:**
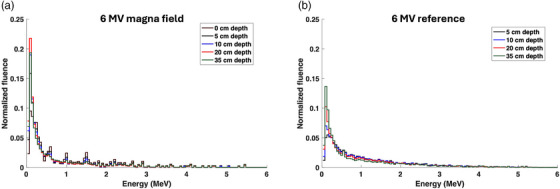
Photon energy spectra at various depths for both (a) 6 MV magna field (165 × 40 × 40 cm^3^ phantom size) and (b) 6 MV reference field.

**FIGURE 4:**
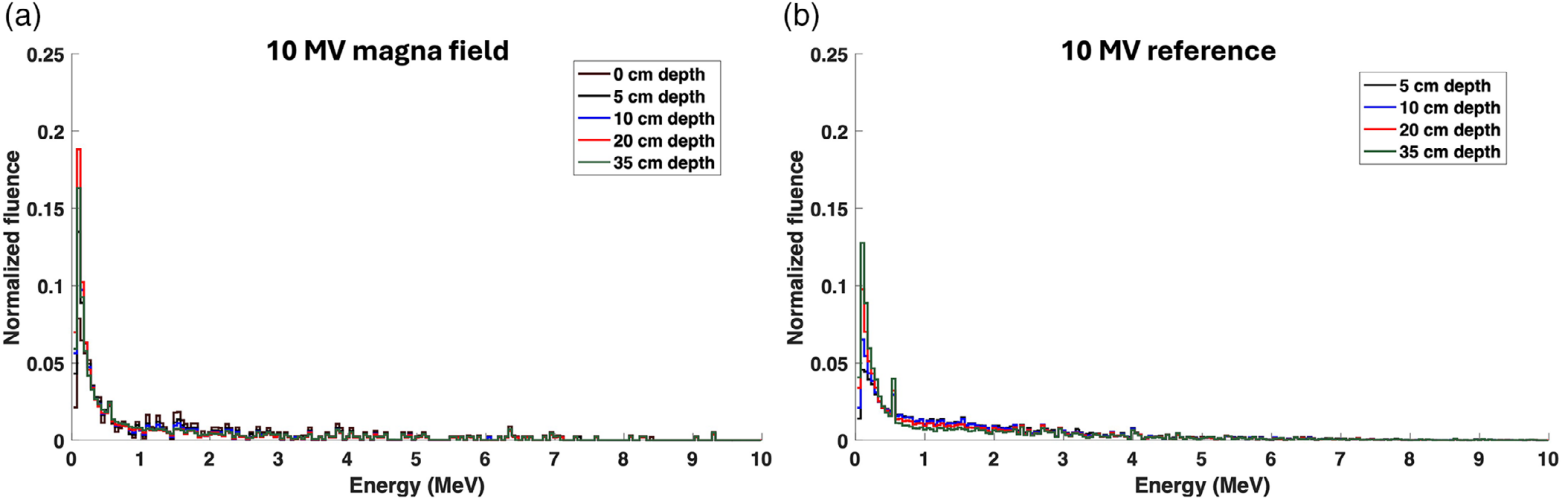
Photon energy spectra at various depths for both (a) 10 MV magna field (165 × 40 × 40 cm^3^ phantom size) and (b) 10 MV reference field.

Figures 3 and 4 show the photon energy spectra at various depths for both 6 and 10 MV photon beams under reference and magna field conditions for the adult‐sized phantom (165 × 40 × 40 cm^3^). A significant increase in the relative bin weight of the lower energy photons was observed with the transition from reference conditions toward magna field conditions. The shift toward lower photon energies is likely due to the increase in Compton‐scattered photons with the increase in phantom scatter and field size. Across both 6 and 10 MV beams for the lowest energy bins, the depth with the highest low‐energy photon fluence switched from 35 cm depth to 20 cm depth with the transition from reference conditions to magna field condition, respectively. For the 10 MV beam shown in Figure 4, the 0.511 MeV photon energy bin weight decreased drastically with the magna field conditions. Since the pair production cross‐section is proportional to the photon energy, a decrease in photon beam energy with the magna field conditions significantly reduces the fluence of 0.511 MeV annihilation photons.

Using the energy spectra calculated under various conditions, the $k_{\mathrm{NR}}$ correction factors are tabulated in Tables 1, 2, 3. It is of note that these correction factors were calculated under the assumption that the magna field and the calibration field have the same nominal photon energy. Due to the varying energy spectra as a function of depth, the correction factors were found to be depth‐dependent. The Farmer ionization chamber demonstrated negligible energy dependence within 0.4%. Therefore, it is recommended to use the same beam quality correction factor, $k_{Q}$, for magna field as the reference condition beam. The LiF TLDs were noted to have a correction factor of up to 2% in the 6 MV beam and 1% in the 10 MV beam. It is well‐known that the TLDs and OSLDs over‐respond in low‐energy photon beams, requiring a correction of less than unity.^16^, ^17^ The alumina‐based OSLDs were found to over‐respond more significantly, leading to correction factors of up to 4.6% and 2.5% in the 6 MV and 10 MV magna fields, respectively. Interestingly, the correction factor for surface dosimetry was found to be minimal, within 1%, across all energies and detector types. With the decrease in phantom size, a trend of reduction in correction factors was noted. To ensure accurate dose measurement, a phantom‐size and depth‐specific correction should be used.

**TABLE 1:** The $k_{\mathrm{NR}}$ correction factors for the Farmer ion chamber for various depths and phantom sizes based on the reference calibration conditions of 100 cm SSD, 10 cm depth, and 10 × 10 cm^2^ field size.

	Near‐infinite phantom 400 × 400 × 40 cm^3^		Adult‐sized phantom 165 × 40 × 40 cm^3^		Small phantom 30 × 30 × 30 cm^3^	
Depth (cm)	6 MV	10 MV	6 MV	10 MV	6 MV	10 MV
0	1.000	1.004	1.000	1.003	1.000	1.000
5	1.000	1.004	1.000	1.003	1.000	1.000
10	1.000	1.003	1.000	1.002	1.000	1.000
20	0.999	1.003	1.000	1.001	1.000	1.000
35	1.000	1.004	1.000	1.001	–	–

Abbreviations: SSD, source‐to‐surface distance.

**TABLE 2:** The $k_{\mathrm{NR}}$ correction factors for LiF TLDs for various depths and phantom sizes based on the reference calibration conditions of 100 cm SSD, 10 cm depth, and 10 × 10 cm^2^ field size.

	Near‐infinite phantom 400 × 400 × 40 cm^3^		Adult‐sized phantom 165 × 40 × 40 cm^3^		Small phantom 30 × 30 × 30 cm^3^	
Depth (cm)	6 MV	10 MV	6 MV	10 MV	6 MV	10 MV
0	0.997	0.998	0.998	0.999	1.000	1.000
5	0.991	0.997	0.992	0.999	0.996	1.001
10	0.986	0.993	0.986	0.997	0.992	1.000
20	0.980	0.989	0.985	0.990	0.994	1.001
35	0.985	0.991	0.990	0.993	–	–

Abbreviations: SSD, source‐to‐surface distance; TLD, thermoluminescent dosimeter.

**TABLE 3:** The $k_{\mathrm{NR}}$ correction factors for alumina OSLDs for various depths and phantom sizes based on the reference calibration conditions of 100 cm SSD, 10 cm depth, and 10 × 10 cm^2^ field size.

	Near‐infinite phantom 400 × 400 × 40 cm^3^		Adult‐sized phantom 165 × 40 × 40 cm^3^		Small phantom 30 × 30 × 30 cm^3^	
Depth (cm)	6 MV	10 MV	6 MV	10 MV	6 MV	10 MV
0	0.990	1.000	0.996	1.000	1.000	1.000
5	0.975	0.990	0.984	0.994	0.993	0.998
10	0.965	0.983	0.972	0.991	0.988	0.994
20	0.952	0.975	0.966	0.980	0.987	0.999
35	0.964	0.981	0.976	0.990	–	–

Abbreviations: OSLD, optically stimulated luminescent dosimeter; SSD, source‐to‐surface distance.

## 4 DISCUSSION

This work investigated the change in energy spectra of 6 and 10 MV photon beams from reference conditions to magna field conditions at extended SSDs. The spectral information was used to derive energy‐dependent correction factors for a Farmer‐type ion chamber, LiF TLDs, and alumina OSLDs. The data provided in this work will advance dosimetry for TBI treatments and allow a more accurate comparison between different detector types.

For a 10 MV photon beam, the work of Garcia et al. reported that the TPR calculated using the corrected PDD curve measured at conventional SSD overestimated the actual TPR by up to 8%.^5^ The spectral softening noted in our study is likely the cause of such an overestimation. Interestingly, this effect was not observed for the 6 MV beam in their work. Contrarily, our results show the opposite effect, where better agreement was noted between the reference TPR and the TBI TPR for the 10 MV beam compared to the 6 MV beam. Since the simulation and measurement conditions between our study and their work differed, additional work with matching conditions is warranted. Although a more drastic reduction in average photon energy was found for the 10 MV beam compared to the 6 MV beam in the magna field conditions, the magnitude of the correction factor is based on the differences in the mass energy absorption coefficient ratios. Since the energy response curve for the TLDs and OSLDs is steeper at lower photon energies, the $k_{\mathrm{NR}}$ correction factor was found to be larger for the 6 MV photon beam. The data provided in this work can be used for reference dosimetry of magna fields given that the depth of the experimental setup is similar to the depths investigated in this study. Additionally, the correction factor at the surface can be used for in vivo dosimetry. Esquivel et al. compared TLDs and OSLDs for dosimetry in a 6 MV TBI magna field and found agreement within 4.6%.^18^ This study found that the energy dependence correction differences can be up to 2.7% between the TLDs and OSLDs. Thus, accounting for these differences may lead to a more accurate comparison between TLDs and OSLDs in the future and reduce uncertainty for these dosimeters. The work of Paloor et al. found large differences in sensitivity correction factors between the reference conditions and the TBI conditions for alumina‐based OSLDs.^19^ Therefore, application of the energy dependence correction factors provided in this study may improve dosimetry for TBI treatments. There are several limitations of this work, including the usage of fixed thickness and material composition of the beam spoiler, providing data for a single extended SSD, and including only a few dosimeters. Nevertheless, the data provided in this study will be useful for improving dosimetry for magna photon fields.

## 5 CONCLUSIONS

This work studied the change in photon energy spectra between reference conditions and magna field conditions used for TBI treatments. The spectral changes were found to be significant, and the dominance of scattered photons in magna fields led to beam softening across all depths. Using the spectral data, energy dependence correction factors were derived for an ion chamber, LiF TLDs, and alumina OSLDs under magna field conditions. The ion chamber was found to exhibit negligible energy dependence, while the correction factors for the TLDs and OSLDs were found to be depth‐dependent with magnitudes up to 2.0% and 4.6%, respectively. Therefore, it is recommended to consider these corrections when performing reference or in vivo dosimetry in TBI magna fields.

## AUTHOR CONTRIBUTIONS

Ahtesham Ullah Khan provided the scientific direction, collected the data, performed data analysis, and prepared the manuscript.

## CONFLICT OF INTEREST STATEMENT

Ahtesham Ullah Khan has nothing to disclose.

## Data Availability

Research data are stored in an institutional repository and will be shared upon request to the corresponding author.
